# Genetic diversity assessment of the indigenous goat population of Benin using microsatellite markers

**DOI:** 10.3389/fgene.2023.1079048

**Published:** 2023-03-17

**Authors:** Habib Rainier Vihotogbe Whannou, Martin Spanoghe, Guiguigbaza-Kossigan Dayo, Dominique Demblon, Deborah Lanterbecq, Luc Hippolyte Dossa

**Affiliations:** ^1^ Ecole des Sciences et Techniques de Production Animale, Faculté des Sciences Agronomiques, Université d’Abomey-Calavi, Cotonou, Benin; ^2^ Laboratory of Biotechnology and Applied Biology, Haute Ecole Provinciale de Hainaut-Condorcet, Hainaut, Belgium; ^3^ Centre International de Recherche-Développement sur l’Elevage en Zone Subhumide (CIRDES), Bobo-Dioulasso, Burkina Faso; ^4^ Haute Ecole Provinciale de Hainaut-Condorcet, Hainaut, Belgium

**Keywords:** *Capra hircus*, molecular genetic characterization, genetic structure, indigenous farm animal genetic resources, phytogeographic zones

## Abstract

Improved knowledge of the diversity within and among local animal populations is increasingly necessary for their sustainable management. Accordingly, this study assessed the genetic diversity and structure of the indigenous goat population of Benin. Nine hundred and fifty-four goats were sampled across the three vegetation zones of Benin [i.e., Guineo-Congolese zone (GCZ), Guineo-Sudanian zone (GSZ), and Sudanian zone (SZ)] and genotyped with 12 multiplexed microsatellite markers. The genetic diversity and structure of the indigenous goat population of Benin were examined using the usual genetic indices (number of alleles Na, expected and observed heterozygosities He and Ho, Fixation index F_ST_, coefficient of genetic differentiation G_ST_), and three different methods of structure assessment [Bayesian admixture model in STRUCTURE, self-organizing map (SOM), and discriminant analysis of principal components (DAPC)]. The mean values of Na (11.25), He (0.69), Ho (0.66), F_ST_ (0.012), and G_ST_ (0.012) estimated in the indigenous Beninese goat population highlighted great genetic diversity. STRUCTURE and SOM results showed the existence of two distinct goat groups (Djallonké and Sahelian) with high crossbreeding effects. Furthermore, DAPC distinguished four clusters within the goat population descending from the two ancestry groups. Clusters 1 and 3 (most individuals from GCZ) respectively showed a mean Djallonké ancestry proportion of 73.79% and 71.18%, whereas cluster 4 (mainly of goats from SZ and some goats of GSZ) showed a mean Sahelian ancestry proportion of 78.65%. Cluster 2, which grouped almost all animals from the three zones, was also of Sahelian ancestry but with a high level of interbreeding, as shown by the mean membership proportion of only 62.73%. It is therefore urgent to develop community management programs and selection schemes for the main goat types to ensure the sustainability of goat production in Benin.

## 1 Introduction

West African countries are characterized in general by high variability in farm animal genetic resources (Molina-Flores et al., 2020). Concerning goat species, Benin’s neighboring countries have remarkably diversified indigenous goat breeds, defined by different ecotypes of West African Dwarf goats (WAD, also named Djallonké goats) found in fifteen West and Central African countries including Togo, Burkina Faso, and Nigeria (Wilson, 1991; Awobajo et al., 2015); Red Sokoto goats in Niger and Nigeria, and a large population of Sahelian goat breeds in Mali, Niger, Burkina Faso and Nigeria (Wilson, 1991). In addition, some exotic goat breeds are also introduced into these countries such as the Boer and Kalahari goats imported into Niger (FAO, 2007). This pool of goat breeds from Benin’s neighboring countries certainly influences the genetic diversity of the indigenous Beninese goat population whose genetic diversity has not been documented to date, unlike that of other African countries like Nigeria (Awobajo et al., 2015; Ojo et al., 2018), Ghana (Ofori et al., 2021), and Burkina Faso (Traoré et al., 2009). Indeed, the previous characterization studies conducted on this species in Benin have been limited to documenting the existing between- and within-species morphological variability (Dossa et al., 2007; Kouato et al., 2021; Whannou et al., 2021) and, habitat suitability modeling of the goat population of Benin under climate change scenarios (Whannou et al., 2022). Thus, there remains a need to determine the genetic diversity within and among this indigenous goat population at the molecular level to optimize their management. Such a study is a response to the Food and Agriculture Organization of the United Nations (FAO, 2012) exhortation to document both phenotypic and molecular diversity of animal genetic resources for better knowledge and definition of policies for their sustainable management. Regarding molecular genetic characterization, different tools, including microsatellite markers, and single-nucleotide polymorphism (SNP) chips have been developed with advances in technology for a better exploration or analysis of the genome. However, although SNPs are highly informative and more nowadays recommended for population genetics studies, their accessibility remains limited, especially in developing countries due to the high costs associated with using this high-definition technology (e.g., cost of chips, high-level infrastructure, and equipment required, and continuous energy power) (Laoun et al., 2020). In contrast, microsatellite markers are less expensive, especially if they are multiplexed, and have demonstrated worldwide their ability to assess diversity in animal population genetics (Ben Sassi-Zaidy et al., 2022). This amply justifies their use in numerous genetic diversity studies conducted in the last years on different species including cattle (Msanga et al., 2012; Gororo et al., 2018; Demir and Balcioğlu, 2019), pigs (Djimènou et al., 2021) and small ruminants (Missohou et al., 2011; Mekuriaw, 2016; Ravimurugan, 2017; Ojo et al., 2018; Dayo et al., 2022). Therefore, microsatellite markers are still highly useful for preliminary studies of the diversity of populations that have never been characterized using molecular tools (Laoun et al., 2020). In such a context, the genetic diversity of the indigenous Beninese goat population could be better documented using microsatellite markers as only phenotype-related information has been reported so far. On the one hand, it should be noted that a review of previous knowledge on the diversity of goat breeds present in Benin has reported the cohabitation of a multitude of West African local breeds such as WAD/Djallonké, Red Sokoto or Maradi, Sahelian (Hounzangbe-Adode et al., 2011; Molina-Flores et al., 2020), and exotic breeds like Alpine and Saanen goats (Hounzangbe-Adode et al., 2011). On the other hand, the most recent study (Whannou et al., 2022) that addressed the phenotypic diversity of the local Beninese goat population revealed the existence of high diversity within and among this indigenous goat population. Moreover, two major groups of goats have been reported within the three vegetation zones (i.e., one group of small individuals mainly in the Guinean-Congolese zone in the South and another group of relatively large goats from the Guinean-Sudanese zone in central Benin to the Sudanese zone in northern Benin).

Hence, this study investigated the genetic diversity and structure of the indigenous goat population in Benin using microsatellite markers to allow a clear identification of breed groups or genetic types and to confirm or refute the phenotypic diversity aforementioned.

## 2 Materials and methods

### 2.1 Sampling procedure

To address the genetic base and structuring of the indigenous goat population of Benin, nine hundred and fifty-four (*n* = 954) randomly sampled goat hair from the three vegetation zones of Benin and used in a previous morphological characterization study (Whannou et al., 2022), were selected from a sample library (*N* = 2,114). These hair samples were selected from unrelated animals using the information provided by goat farmers on their animals. Some characteristics of these vegetation zones i.e., humidity index, soil characteristics, and predominant vegetation, can be found in Whannou et al. (2022). The vegetation zones are further subdivided into phytogeographic zones. The minimum sample size was about 286 individuals per vegetation zone and 92 individuals per phytogeographic zone. These samples were labeled, packaged, and transported to the laboratory in Belgium (CARAH, Ath, Hainaut) for DNA extraction and genotyping.

### 2.2 DNA extraction and genotyping

DNA was extracted from hair samples following the standard instructions described for the Qiagen DNeasy Blood and Tissue Kit used. Each DNA sample was then quantified using a NanoDrop ND-3300 fluorospectrometer device (Thermo Scientific; Waltham, MA, United States).

The genotyping analysis was performed with 15 μL of template DNA using the multiplex kit of 12 microsatellite markers and the PCR protocol developed by Spanoghe et al. (2022). The fragment lengths of the PCR products were estimated with the GeneMapper Software 6.0 (Applied Biosystems). They were then used to construct a genotypic dataset for statistical analyses.

### 2.3 Statistical analysis

#### 2.3.1 Genetic diversity assessment

The number of alleles (Na), the effective number of alleles (Nae), observed (Ho) and expected (He) heterozygosities, and Polymorphic Information Content (PIC) of each microsatellite marker were first estimated from the dataset (*n* = 954) using the Cervus software v 3.0 (Kalinowski et al., 2007). These statistics were addressed to assess the performance of the loci and to describe the genetic diversity of the Beninese goat population. F-statistic indices (F_IS_, F_ST_, F_IT_) (Wright, 1969; Weir and Cockerham, 1984), the coefficient of gene differentiation (G_ST_), and Nei’s genetic distance (Nei, 1978) were then computed using the program SPAGeDi 1.5 days (Hardy and Vekemans, 2002) to assess the genetic variability existing within (intra-) and among (inter-) vegetation zones.

Additionally, an analysis of molecular variance (AMOVA) was performed to assess the partition of genetic variation between (inter-) and within (intra-) the goat groups (Excoffier et al., 1992; Paradis, 2010).

#### 2.3.2 Genetic clustering analyses of the goat population under study

Three methods were used to estimate the genetic clustering of the goat samples of Benin and their genetic relationship.

First, the genetic structure of the indigenous goat population was analyzed using the Bayesian admixture approach in the STRUCTURE software 2.3.4 (Pritchard et al., 2000). The ancestry proportion was inferred from the genotypic dataset using correlated allele frequencies, a burn-in period of 50,000 iterations followed by 100,000 Markov Chain Monte Carlo (MCMC) for each number of possible clusters (K). As genotyping information for the assumed parent population was not available, we hypothesized K unknown populations of parents with k varying from 1 to 10, and three independent replicates (Negrini et al., 2012). The probable number K of ancestral populations and substructures was identified according to Evanno et al. (2005) and the obtained posterior probability values (Pritchard et al., 2000). The representation of the data was then performed using Structure Plot (Ramasamy et al., 2014), and the geographic distribution of the main genotype of goats across the vegetation and phytogeographic zones of Benin was mapped using the Q matrix out-put.

Second, the non-linear relationships of the genotypic data were estimated using the Self-Organizing Map (SOM) method (Kohonen, 1982; 2001) under unsupervised learning rules and based on the model of vegetation zones of Benin (See Spanoghe et al., 2020 for a full description of the method).

Third, Discriminant Analysis of Principal Components (DAPC) (Jombart, 2008; Jombart et al., 2010) was applied to the genotypic dataset to infer the relationship of goat individuals, while maximizing among-group variation and minimizing within-group variation. Unsupervised k-means clustering was first used through the “*find.clusters*” function of the R package *adegenet* version 2.1.1 (Jombart, 2008) to estimate the probable number of clusters existing in the Beninese goat population. The number of clusters (K) was then defined after a comparison of Bayesian Information Criterion (BIC) values (Jombart, 2008; Jombart and Ahmed, 2011). The resultant clusters were plotted in a scatterplot after the determination of the number of principal components (PCs) with associated linear discriminants (LD) using the cross-validation function “*Xval.dapc*” in the R package *adegene*t.

Finally, the genetic variation existing within and among the inferred goat groups from genetic clustering with DAPC was estimated using the genetic parameters previously calculated in the first section of Statistic analysis (i.e., Genetic diversity assessment).

## 3 Results

### 3.1 Genetic diversity of the indigenous goat population from Benin

The different genetic indices Na, He, Ho, PIC, F_IS_, F_ST_, F_IT_, and G_ST_ estimated from the Beninese goat dataset are presented in Table 1. Overall, 135 alleles were identified in the dataset with the multiplex of 12 microsatellite markers, with an average of 11.25 alleles per locus. The lowest Na (4) was recorded for the ILSTS5 locus, and the highest Na (23) was detected for the MAF065 locus. The average values of He and Ho were 0.66 and 0.69, respectively. The PIC ranged from 0.14 (ILSTS5) to 0.80 (SCRSP9 and CSRD247) with an average value of 0.66. The mean values of F_ST_, F_IT_, F_IS_, and G_ST_ were 0.012, 0.047, 0.035, and 0.012 respectively.

**TABLE 1:** Genetic diversity indices calculated for 12 SSR markers in 954 goat datasets sampled in the three vegetation zones of Benin.

SSR markers	Na	Scale	Ho	He	PIC	F_IT_	F_ST_	F_IS_	G_ST_
ILSTS11	10	264–282	0.48	0.49	0.44	0.008	0.002	0.006	0.002
ILSTS5	4	184–192	0.14	0.15	0.14	0.093	0.011	0.083	0.010
MAF065	23	118–183	0.76	0.81	0.79	0.068	0.011	0.056	0.010
MCM527	6	153–168	0.69	0.73	0.68	0.065	0.016	0.050	0.015
SCRSP9	14	117–147	0.80	0.82	0.80	0.032	0.011	0.021	0.010
TCRVB6	14	222–255	0.66	0.68	0.65	0.034	0.009	0.025	0.009
INRA023	13	195–218	0.76	0.80	0.77	0.057	0.031	0.027	0.030
OARFCB20	11	94–119	0.71	0.76	0.73	0.069	0.017	0.054	0.016
OARFCB48	11	151–171	0.74	0.78	0.74	0.045	0.006	0.039	0.006
BM8125	10	111–131	0.74	0.76	0.72	0.016	0.006	0.010	0.006
CSRD247	12	220–249	0.78	0.82	0.80	0.056	0.014	0.043	0.013
INRA063	7	172–184	0.66	0.68	0.63	0.042	0.007	0.035	0.007
Mean	11.25	—	0.66	0.69	0.66	0.047	0.012	0.035	0.012

Na, Number of alleles per marker scale; Ho, observed heterozygosity; He, expected heterozygosity; PIC, polymorphic information content; F_IT_, intra-class correlation coefficients of allelic states for gene copies within individuals relative to all populations; F_ST_, gene copies within populations relative to all populations; F_IS_, gene copies within individuals relative to a population; G_ST_, Nei’s coefficient of gene variation.

The AMOVA results (Table 2) show that only 2.11% of the genetic variation of the Beninese goat population was observed between vegetation zones; the highest genetic variation (97.89%) resided within vegetation zones. Using the vegetation zones as a model of structuring (Table 3), Na ranged from 9.08 (GCZ) to 10.08 (GSZ), with a mean value of 9.55. SZ and GSZ showed the highest values of He (0.70 and 0.69, respectively) and Ho (0.67 for both vegetation zones) as well as the highest F_IS_ values (0.05 and 0.04, respectively). The highest pairwise F_ST_ (0.021) and Nei’s genetic distance (0.047) were recorded between GCZ and SZ, whereas the lowest F_ST_ (0.006) and Nei’s genetic distance (0.013) were observed between GSZ and SZ (Table 3). However, the pairwise F_ST_ and Nei’s genetic distances estimated between GCZ and GSZ were also low and seemed less different from those recorded between GSZ and SZ (Table 3).

**TABLE 2:** Analysis of molecular variance (AMOVA) of the 954 goats within and among the three vegetation zones of Benin.

	Degree of freedom	Sum of square	Variance component	Percentage of variation	Phi-value (φ)	Gene flow (Nm)
Between vegetation zones	2	175.28	0.24	2.11	0.02	315
Within vegetation zones	951	10,679.13	11.23	97.89		

**TABLE 3:** Genetic diversity parameters of the 954 goats within and among the three vegetation zones of Benin.

Vegetation zones^a^	Within vegetation zones parameters						Between vegetation zones parameters^b^		
	n	Na	Nae	He	Ho	F_IS_	GCZ	GSZ	SZ
GCZ	377	9.08	3.65	0.67	0.65	0.03	—	0.015	0.047
GSZ	286	10.08	3.97	0.69	0.67	0.04	0.007	—	0.013
SZ	291	9.50	3.98	0.70	0.67	0.05	0.021	0.006	—
Mean	—	9.55	3.87	0.69	0.66	0.04			

n = number of individuals analyzed, Na = number of alleles, Nae = effective number of alleles, He = expected heterozygosity, Ho = observed heterozygosity, F_IS_ = individual inbreeding coefficient.

^a^ GCZ: Guineo-Congolese zone, GSZ: Guineo-Sudanian zone, SZ: Sudanian zone.

^b^ F_ST_ below the diagonal and Nei’s genetic distance above the diagonal.

### 3.2 Genetic structure of the indigenous goat population from Benin

**FIGURE 1:**
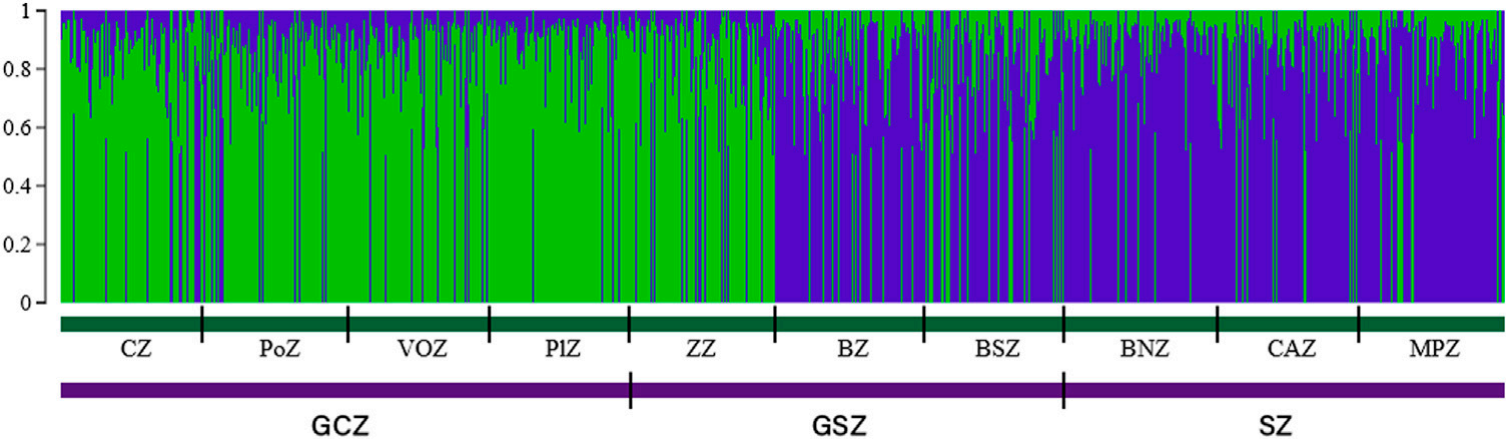
Goat population structure determined by STRUCTURE 2.3. Estimated histogram of the population structure with two ancestral populations (*K* = 2). Each vertical bar represents one individual in the population based on the percentage of group membership, into the 2 inferred subpopulations.

**FIGURE 2:**
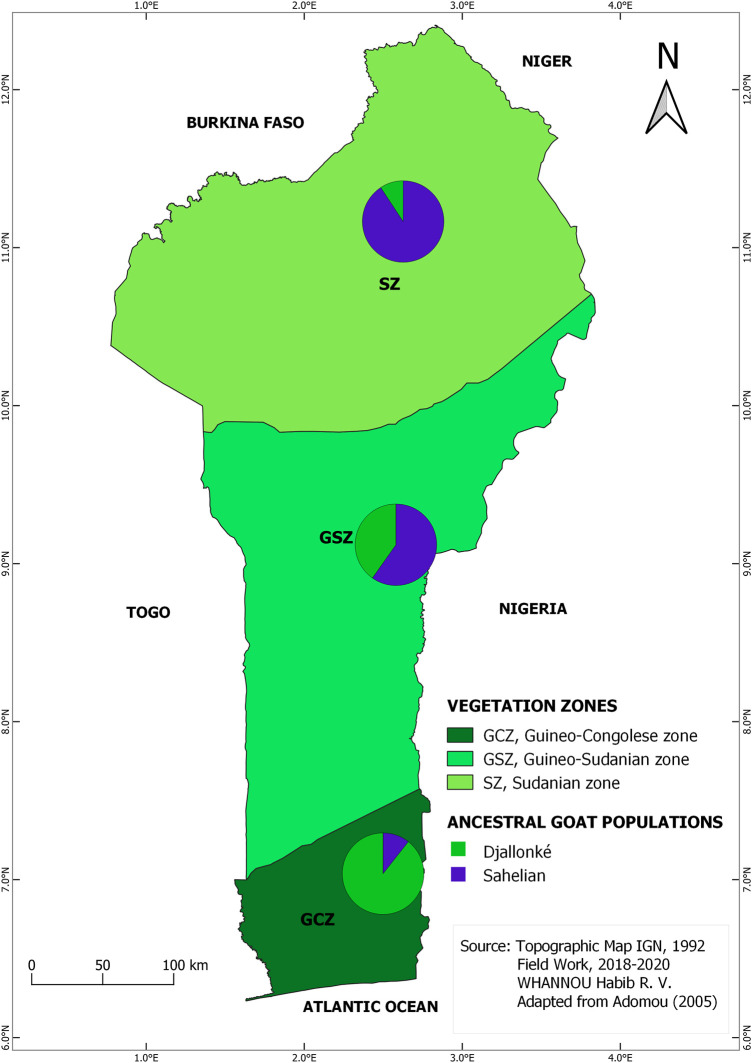
Map of the spatial distribution of the two inferred ancestral populations based on membership assignment from the population structure analysis following vegetation zones pattern.

**FIGURE 3:**
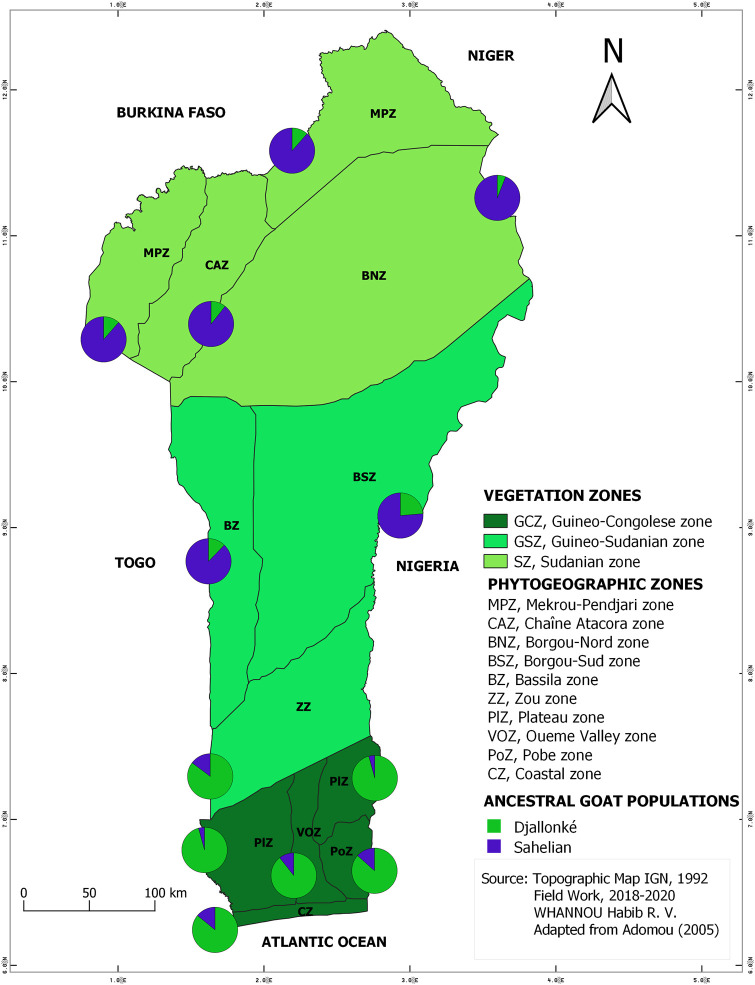
Map of the spatial distribution of the two inferred ancestral populations based on membership assignment from the population structure analysis following vegetation and phytogeographic zones patterns.

The STRUCTURE results suggested the best grouping number (*K* = 2) based on the highest delta K value (53.17) resulting from the data (Supplementary Table S1; Supplementary Figure S1). The indigenous goat population of Benin was therefore composed of two ancestral genetic groups with different ancestry proportions of individuals. Overall, 50.20% of the population analyzed was estimated as Djallonké ancestry, whereas 49.80% was of Sahelian ancestry (Supplementary Table S2). The individuals’ membership proportion revealed some admixture, indicating that individuals share different proportions of the two distinct ancestral goat populations (i.e., Djallonké and Sahelian) (Figure 1). Considering that individuals presenting a membership proportion of more than 50% for ancestry population 1 (in green) were mainly ancestry of Djallonké and those that presented a membership proportion of more than 50% for ancestry population 2 (in blue) were mostly of Sahelian ancestry, it appeared that individuals from GCZ were predominantly of Djallonké ancestry, those of SZ were of Sahelian ancestry, whereas the GSZ predominantly included Sahelian genotypes (Figure 2). However, according to a smaller subdivision than vegetation zones i.e., the phytogeographic zones (Figure 3), a predominance of Djallonké ancestry was noted in the four phytogeographic zones of GCZ (i.e., CZ Coastal zone, PoZ Pobe zone, PlZ Plateau zone, and VOZ Oueme Valley zone), and the phytogeographic zone of the GSZ closest to the GCZ (i.e., ZZ Zou zone). In contrast, the two other phytogeographic zones of the GSZ (i.e., BZ Bassila zone, and BSZ Borgou-Sud zone) and the phytogeographic zones of the SZ (i.e., BNZ Borgou-Nord zone, CAZ Chaîne Atacora zone, and MPZ Mekrou-Pendjari zone) gathered mostly goats with predominant Sahelian ancestry (Figure 3).

**FIGURE 4:**
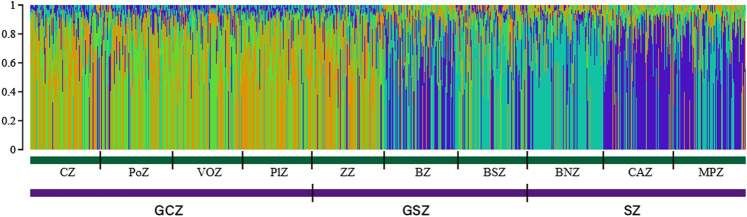
Goat population structure determined by STRUCTURE 2.3. Estimated histogram of the population structure with two ancestral populations (*K* = 4). Each vertical bar represents one individual in the population based on the percentage of group membership, into the 4 inferred subgroups.

Furthermore, when the log-likelihood of the data Ln P(D) was plotted against K, the average log-likelihood of the data Ln P(D) increased up to *K* = 4, followed by a serrated decrease to *K* = 9 (Supplementary Figure S2). The run with the highest Ln P(D) was thus observed at *K* = 4 suggesting a structuration of the goat population under study into four subpopulations. The STRUCTURE plot for *K* = 4 (Figure 4) indicated the existence of two goat subpopulations of Djallonké distributed from the humid zone of South Benin (GCZ) to the first phytogeographic zone (i.e., ZZ) of the transitional vegetation zone in Central Benin (GSZ). Two other subpopulations of goats sharing mostly Sahelian ancestry were observed from the remaining two phytogeographic zones of the GSZ (i.e., BZ, and BSZ) to the drier Sudanian vegetation zone (SZ) in North Benin (Figure 4).

**FIGURE 5:**
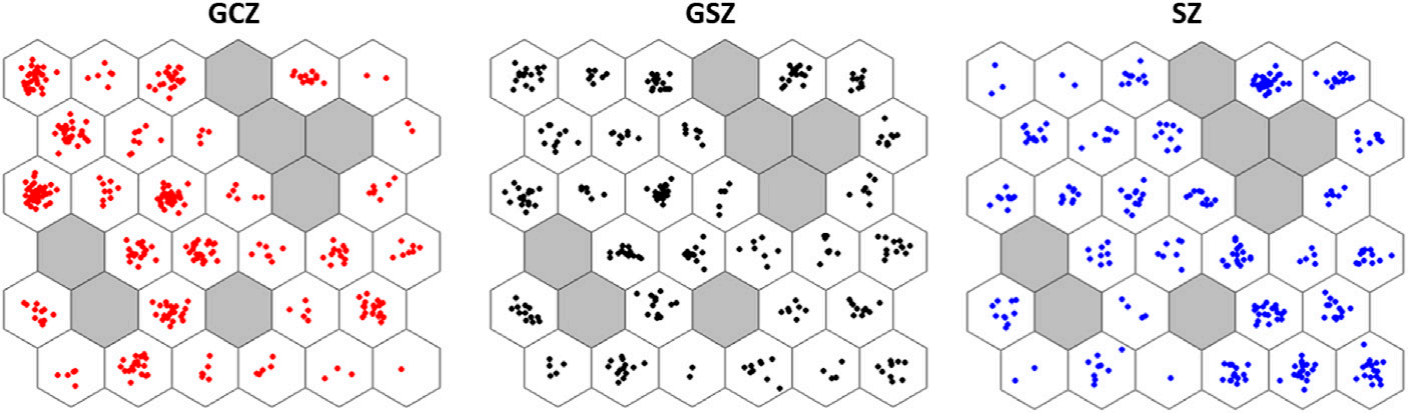
Distribution of the genotyped goats on the SOM network according to the assignment of each of the vegetation zone groups. Each colored dot corresponds to a goat individual. The plots express individual’s assignment by emphasizing vegetation zone models where GCZ: Guineo-Congolese zone, GSZ: Guineo-Sudanian zone, and SZ: Sudanian zone.

SOM analysis showed the neural assignment of individuals on the network (Figure 5). The structuring of the goat population in the different vegetation zones seems rather diffuse and scattered since all neurons are occupied whatever the vegetation zones. Nevertheless, individuals from GCZ were mostly concentrated in left neurons in the network, while SZ individuals were mostly clustered in right neurons in the network. GSZ individuals, although widely distributed across grid neurons, appeared more concentrated in left and some upper right neurons.

**FIGURE 6:**
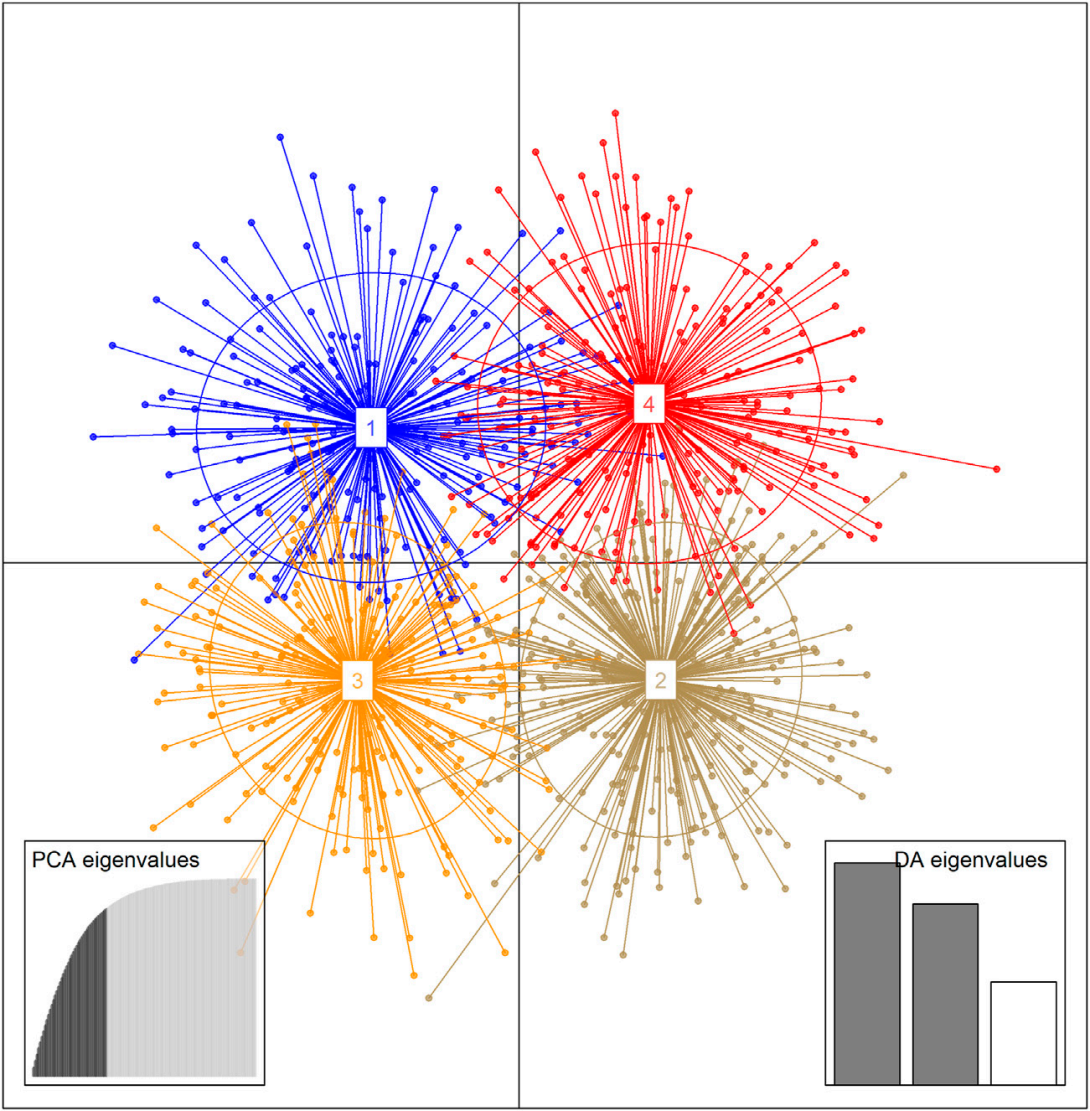
Scatterplot of the first two Linear Discriminants (LD) showing genetic clusters for 954 indigenous goat sampled in the three vegetation zones of Benin applying unsupervised Discriminant Analysis of Principal Components (DAPC). Each ellipse represents *a priori* cluster and each dot an individual.

The results of the unsupervised K-means clustering applied to the dataset prior to DAPC showed BIC values that decreased between *K* = 2 and *K* = 8 where they reach the lowest value of BIC (Supplementary Figure S3). Thus, any K value between 2 and 8 could be considered as the number of clusters present in the Beninese goat population. However, when plotting each probable clustering from 2 to 8, a distinction of goat clusters was first observed at *K* = 4. Indeed, all the previous K (i.e., *K* = 5, *K* = 6, *K* = 7, and *K* = 8) showed many overlaps and representation of four probable goat groups in the dataset (Supplementary Figures S4–S7). Thus, four genetic clusters were considered the most probable groups fitting the structure of the indigenous goat population from Benin. DAPC analysis was carried out to assess the sub-clusters at *K* = 4. After the cross-validation step, the 45 first PCs (85% of variance conserved) of PCA and two discriminant eigenvalues were retained. The resulting scatterplot (Figure 6) showed the separation between clusters 1 and 3 (which consisted mainly of individuals from GCZ) and clusters 2 and 4 (which consisted mainly of individuals from SZ and GSZ) concerning LD1. Furthermore, clusters 1 and 3 were distinct from clusters 2 and 4, respectively, with respect to LD2. Table 4 presents the composition of the goat clusters identified within the three vegetation zones of Benin.

**TABLE 4:** Genetic clusters inferred for 954 goats from the three vegetation zones of Benin by applying the unsupervised discriminant analysis of principal components (DAPC).

Vegetation zones	Clusters			
	C1 (*n* = 208)	C2 (*n* = 292)	C3 (*n* = 239)	C4 (*n* = 215)
GCZ	139	85	120	33
GSZ	54	89	64	79
SZ	15	118	55	103

GCZ, Guineo-Congolese zone; GSZ, Guineo-Sudanian zone; SZ, Sudanian zone.

Additionally, when comparing the individuals of inferred DAPC clusters with membership proportions of ancestral goat groups resulting from STRUCTURE (Table 5), it was estimated that individuals of cluster 1 (C1) and cluster 3 (C3) were mainly of Djallonké ancestry with the mean proportion of 73.79% and 71.18%, respectively, while goats of cluster 2 (C2) and cluster 4 (C4) were of Sahelian ancestry with a mean proportion of 62.73% and 78.65%, respectively.

**TABLE 5:** Mean, minimum, and maximum of ancestral proportions (estimated in structure) for the clusters inferred with the unsupervised clustering in DAPC in the sampled goat population (*N* = 954).

Ancestral populations	Statistics	Clusters			
		C1	C2	C3	C4
		(*n* = 208)	(*n* = 292)	(*n* = 239)	(*n* = 215)
Djallonké goat	Mean (%)	73.79	37.27	71.18	21.35
	Minimum (%)	7.00	2.20	3.90	2.30
	Maximum (%)	98.10	96.30	97.70	94.60
Sahelian goat	Mean (%)	26.21	62.73	28.82	78.65
	Minimum (%)	1.90	3.70	2.30	5.40
	Maximum (%)	93.00	97.80	96.10	97.70

### 3.3 Genetic diversity of the estimated DAPC clusters

Table 6 presents the genetic variation within and among the estimated DAPC clusters. Na within the four inferred DAPC clusters ranged between 8.92 (C1) and 10.08 (C2) with an average value of 11.25. Nae ranged between 3.43 (C1) and 3.77 (C2 and C4) with a mean value of 3.93. However, clusters C4 and C2 showed high degrees of He (0.69 and 0.68, respectively) and Ho (0.67 for both clusters) compared with C1 (He = 0.64, Ho = 0.65) and C3 (He = 0.65, Ho = 0.65) that recorded the lowest values. F_IS_ recorded within the clusters ranged between −0.01 (C1) and 0.03 (C4) with a mean value of 0.04. Considering the F_ST_ values recorded between the inferred DAPC clusters, the highest F_ST_ value (0.06) was estimated between C3 and C4. A similar F_ST_ value (0.04) was recorded between the pairs (C1-C3, C1-C4, C2-C3, and C2-C4). Additionally, a low Nei’s genetic distance was recorded between C1 and C3, whereas a high distance was estimated between C3 and C4, but smaller than that recorded between C1 and C3.

**TABLE 6:** Genetic diversity parameters of the inferred clusters from the Beninese goat population (*N* = 954).

Clusters	Within clusters						Between clusters^a^			
	n	Na	Nae	He	Ho	F_IS_	C1	C2	C3	C4
C1	208	8.92	3.43	0.64	0.65	−0.01		0.10	0.08	0.10
C2	292	10.08	3.77	0.68	0.67	0.01	0.05		0.09	0.09
C3	239	9.25	3.51	0.65	0.65	0.002	0.04	0.04		0.14
C4	215	9.75	3.77	0.69	0.67	0.03	0.04	0.04	0.06	
Mean		11.25	3.93	0.69	0.66	0.04				

n = number of individuals analyzed, Na = number of alleles, Nae = effective number of alleles, He = expected heterozygosity, Ho = observed heterozygosity, F_IS_ = individual inbreeding coefficient.

^a^ F_ST_ below the diagonal and Nei’s genetic distance above the diagonal.

## 4 Discussion

This study constitutes the first one performed on the genetic diversity within the goat population of Benin. All the microsatellite loci used in this study were informative because they recorded at least 4 alleles (Barker et al., 2001) and most of them obtained high PIC values (PIC>0.50) (Arora et al., 2010; Botstein et al., 1980). Regarding the genetic diversity indices estimated, the mean values of Na (11.25), He (0.69), Ho (0.66), and PIC (0.66) recorded in this study revealed a high genetic diversity within the goat population of Benin (Kumar et al., 2009; Jawasreh et al., 2018; Mihailova, 2021). The average Ho (0.66) obtained is higher than that reported for the Ardi goat from the Saudi Arabia Kingdom (0.55) (Aljumaah et al., 2012), the Nigerian West African Dwarf goat (0.60) (Awobajo et al., 2015), and the Nigerian indigenous goat population (0.61) (Ojo et al., 2018). However, it is lower than the mean Ho value (0.84) reported for four Algerian goat breeds (Tefiel et al., 2018). The mean value of PIC (0.66) obtained in this study was lower than values reported in Indian goat breeds (0.77) (Dixit et al., 2012), in Nigerian West African Dwarf goats (0.69) (Awobajo et al., 2015), and Algerian goat breeds (0.93) (Tefiel et al., 2018). Although the Beninese goat population appeared diverse, the low F_IS_ (0.035) and F_IT_ (0.047) values recorded suggest some inbreeding events in this population (Tolone et al., 2012). Indeed, a positive F_IS_ value is generally considered as an indicator of heterozygosity deficit compared with Hardy-Weinberg equilibrium (Tefiel et al., 2018). Nevertheless, obtained values of F_IS_ and F_IT_ were lower than those (F_IS_ = 0.090, F_IT_ = 0.180) reported by Awobajo et al. (2015) (F_IS_ = 0.105, F_IT_ = 0.129) by Ojo et al. (2018) (F_IS_ = 0.035, F_IT_ = 0.063) by Traoré et al. (2009) in Burkina Faso goats, and to (F_IS_ = 0.057, F_IT_ = 0.102) reported by Tefiel et al. (2018) in the four Algerian goat breeds. This highlights the diversity of indigenous goat populations in Africa, and probably reflects the difference in the management of goat resources from one country to another.

The mean value of F_ST_ (0.012) obtained in this study was inferior to 0.05, indicating a very low genetic differentiation in the goat population of Benin. The coefficient of gene differentiation (G_ST_) obtained with a mean value of 0.012 confirmed the limited genetic differentiation between vegetation zones. The result of the AMOVA applied to the dataset using vegetation zones as a like-effect of variation also confirmed this limited genetic differentiation. Therefore, the genetic differentiation of the Beninese goat population is intraspecific diversity, thus mainly due to the diversity between individuals within vegetation zones. The lack of genetic differentiation observed between vegetation zones is probably due to different factors including the proximity of production areas, similar extensive breeding practices in the different vegetation zones, but especially the gene flow that occurred between individuals of the main goat groups in the past. A similar finding has been reported by Tolone et al. (2012). The proximity of the breeding areas certainly favors the continuous exchange of breeds through the market system and other mechanisms developed by the different actors of the goat value chain, such as gifts. Moreover, the extensive breeding practices developed by goat breeders (notably the non-control of reproduction in most breeding areas) in all vegetation zones are probably also levers of diversity in the Beninese goat population and therefore favor the low genetic differentiation observed. In comparison to other studies, the average F_ST_ over loci (0.012) estimated in the Beninese goat population is lower than the value obtained in goat populations of Burkina Faso (0.035) (Traoré et al., 2009), Nigeria (0.10) (Awobajo et al., 2015) and (0.030) (Ojo et al., 2018), and Algeria (0.048) (Tefiel et al., 2018). Therefore, the indigenous goat population of Benin is less differentiated than those of other African countries.

The high mean values of Na, He, Ho, and F_IS_ obtained in GSZ and SZ goat subpopulations when measuring the genetic diversity existing within and among the vegetation zones, underline that the goats of these vegetation zones are very diverse, but some individuals from these zones are also inbred. In a similar study, Tolone et al. (2012) also recorded high He and Na values within subpopulations or breed groups, with high F_IS_, and concluded a high genetic diversity within these subpopulations or breed groups. Furthermore, the highest values of pairwise F_ST_ and Nei’s genetic distance recorded between GCZ and SZ confirm that goats from these two vegetation zones are genetically different. In contrast, the lowest values of F_ST_ and Nei’s genetic distance obtained between GSZ and SZ suggest that goats from these zones are genetically close. However, some goats from GSZ would be also genetically closer to GCZ individuals, and their genetic proximity seems similar to that observed between GSZ and SZ, as shown by their near similarity between the indices of genetic differentiation and the genetic distance of Nei’s (Table 3). These results suggest that GSZ is an intermediate subpopulation of goats with a high gene flow. In a recent study of phenotypic diversity, Whannou et al. (2022) stated that GCZ grouped mainly small-size goats, namely, Djallonké, whereas large and intermediate goat types (i.e., Sahelian and crossbreed goats) predominated in SZ and GSZ. Moreover, these authors argued that GSZ may be considered an interbreeding zone. Therefore, the current genetic findings agree to some extent with previous results on phenotypic diversity.

The investigation of the genetic structure of the Beninese goat population using three different methods (STRUCTURE, SOM, and DAPC) confirmed the aforementioned results. First, the STRUCTURE results confirmed the widely accepted existence of two existing ancestral populations of goats in Benin (Meyer, 2002; Dossa et al., 2007; Hounzangbé-Adote et al., 2011) with gene flows between these populations, as suggested by the most probable value of *K* = 2 groups and proportions of individuals’ assignment. Moreover, the STRUCTURE results showed that goats in GCZ and SZ were genetically more distant than that observed between GSZ and SZ. Indeed, GSZ grouped the two distinct goat genotypes. Second, SOM results supported the lower genetic differentiation existing between individuals from vegetation zones and suggested a distinction between goats from GCZ and those from SZ, but the closeness of individuals from GSZ to those of the two other distinct zones. Finally, the DAPC results that reveal the existence of four goat genetic clusters (C) in Benin according to both vegetation and phytogeographic zones, confirm the geographic distribution of goat types in Benin as previously defined based on morphology (Whannou et al., 2022). These results also show that the two main ancestral goat populations are highly crossed, with a critical purity degree of only 70% for the purest subpopulations (i.e., C1, C3, and C4) (Table 5). Considering these results, there is a risk of losing part of genetic diversity if no breeding policy is defined to maintain some pure individuals of the main goat types. Moreover, there are no reliable updated data on the population size of the different goat genetic types identified due to the lack of organization in the goat farming sector in Benin. As a result, the sustainability of goat resources in Benin would be threatened if crossbreeding practices continue anarchically on farms without measures being taken to conserve the predominant genetic types. New management policies for goat keeping in Benin are therefore essential to ensure their sustainable use and to face the challenges of current and future climate and societal changes. To achieve this, an inventory of goat genetic resources should be organized at the national level together with the elicitation of goat farmers’ preferences for goat breeds and production objectives and will allow the establishment of guidelines for maintaining the existing diversity within the goat population in Benin.

## 5 Conclusion

This study provides valuable data on the genetic diversity and structure of the indigenous goat population of Benin and fairly confirms the phenotypic diversity observed within this population. Indeed, the results highlighted the presence of two ancestral genetic groups of goats in Benin with a high level of interbreeding, particularly in GSZ. However, although the indigenous goat population of Benin is highly diverse, the pressure of poorly planned and controlled crossbreeding might threaten the sustainability of goat farming systems. With the current pressure of climate and societal changes, any threat to local goat resources should be prevented more than ever. Measures for the conservation and sustainable management of indigenous goat resources need to be taken involving the farmers who are the owners of these animal genetic resources. For instance, sensitization and training sessions could be organized to raise the awareness of farmers on the need to maintain farm animal genetic resources, to show them the importance and necessity of monitoring and organizing reproduction in their herds, and to remind them or strengthen their knowledge of the qualities of local breeds such as the trypanotolerance and prolificacy of the Djallonké goats with a view to establishing purebred breeding. In addition, the Beninese government should, in the long term, introduce breeding laws and policies to control the movement of animals both at the borders and within Beninese localities. Finally, conservation programs for the local breeds should be urgently set up.

## Data Availability

The original contributions presented in the study are included in the article/Supplementary Materials, further inquiries can be directed to the corresponding author.
