# Supplementary material for: Genetic diversity assessment of the indigenous goat population of Benin using microsatellite markers
Source: Front Genet. 2023 Mar 17;14:1079048. doi: 10.3389/fgene.2023.1079048 (PMC10064012; doi:10.3389/fgene.2023.1079048)
Supplement: Supplementary file 1 [file DataSheet2.docx]

Supplementary Material

# Supplementary Figures


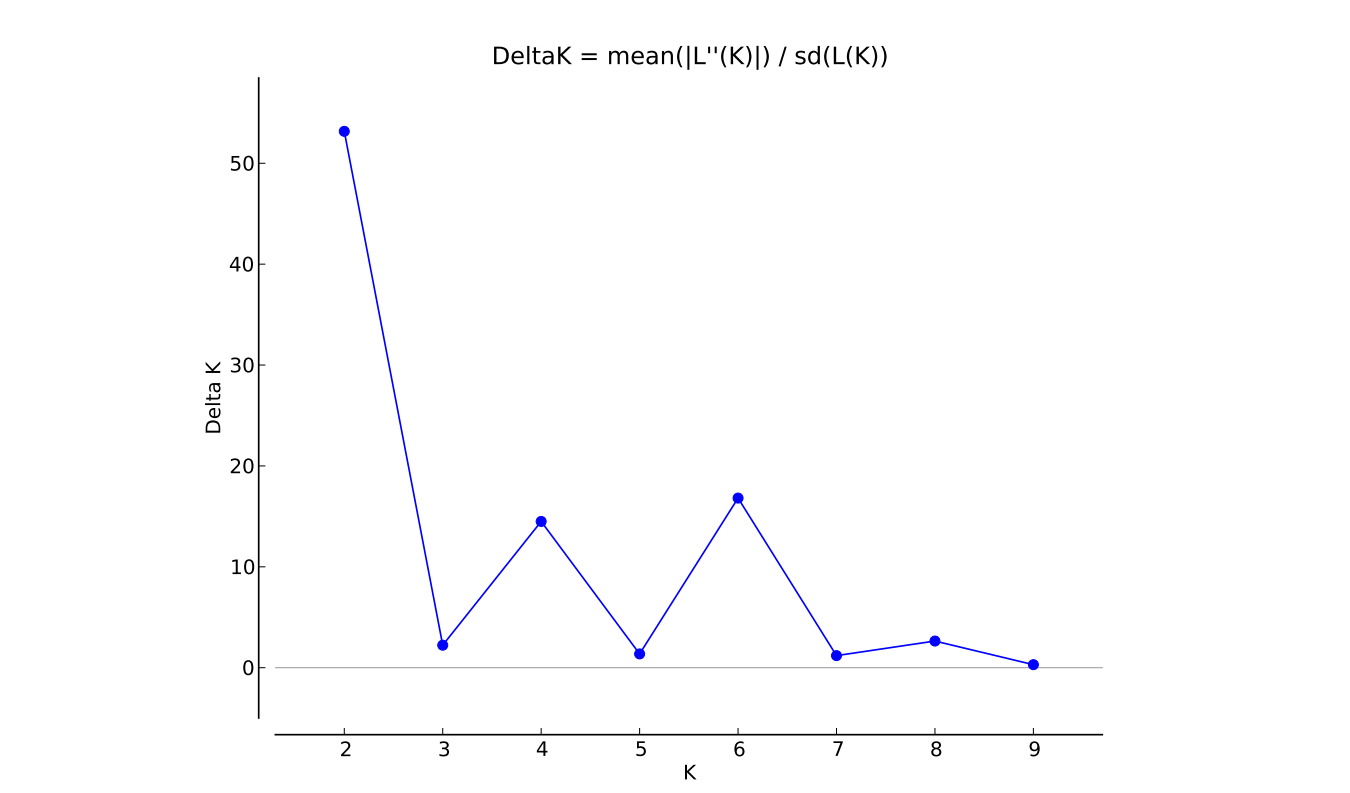


**Supplementary Figure 1.** Graphical representation of posterior probability of the data across different potential numbers of ancestral populations following the method of Evanno et al. (2005).

**
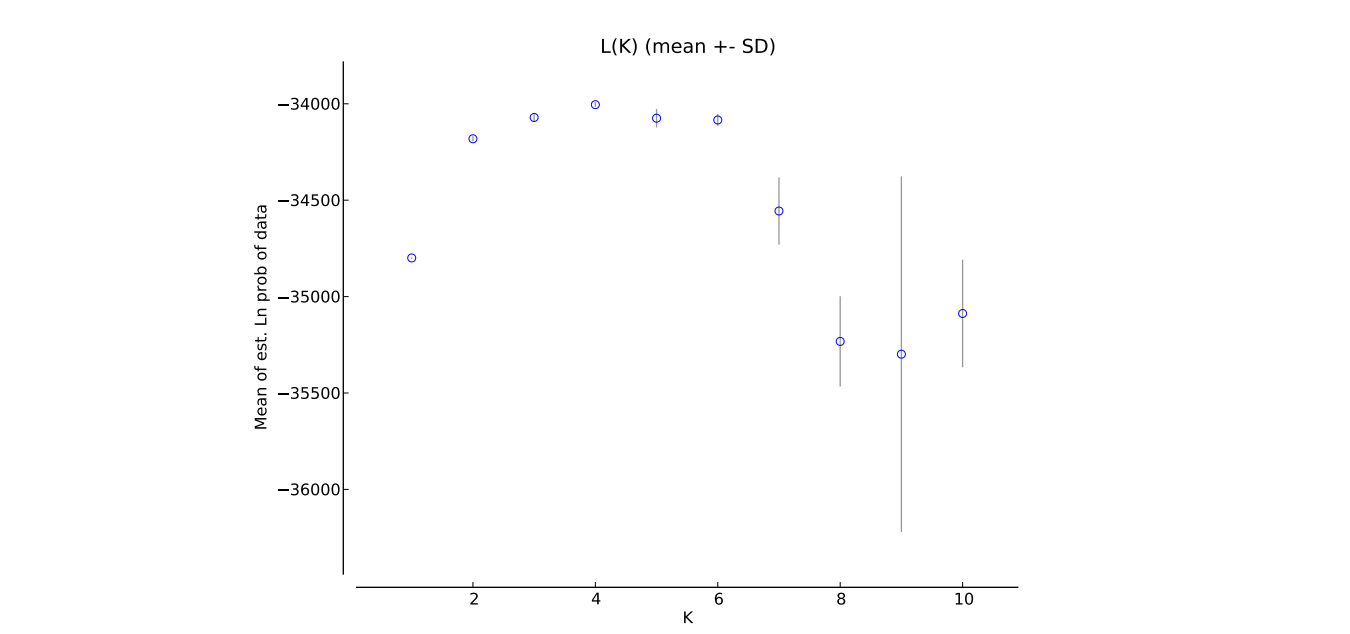
**

**Supplementary Figure 2.** Graphical representation of mean LnP(K) following the stability of replicate runs for each assessed K.


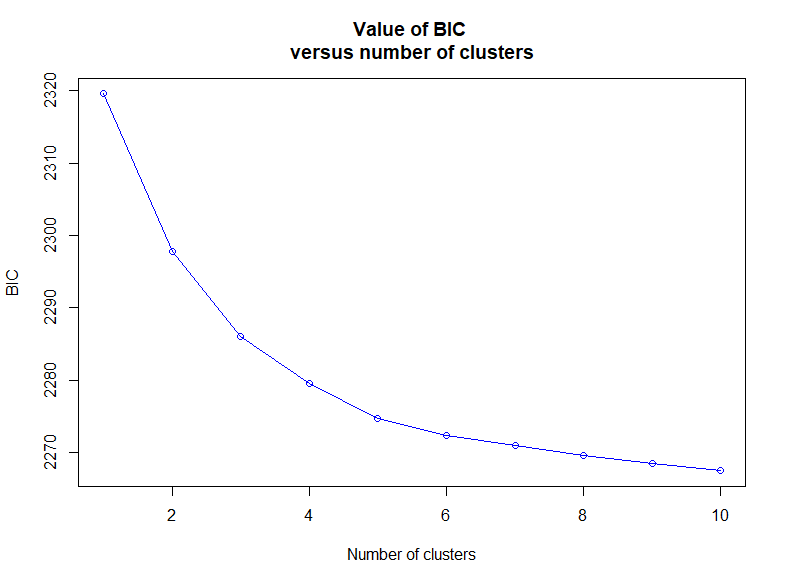


**Supplementary Figure 3.** Inference of the number of clusters in the dataset of 954 indigenous goats from Benin


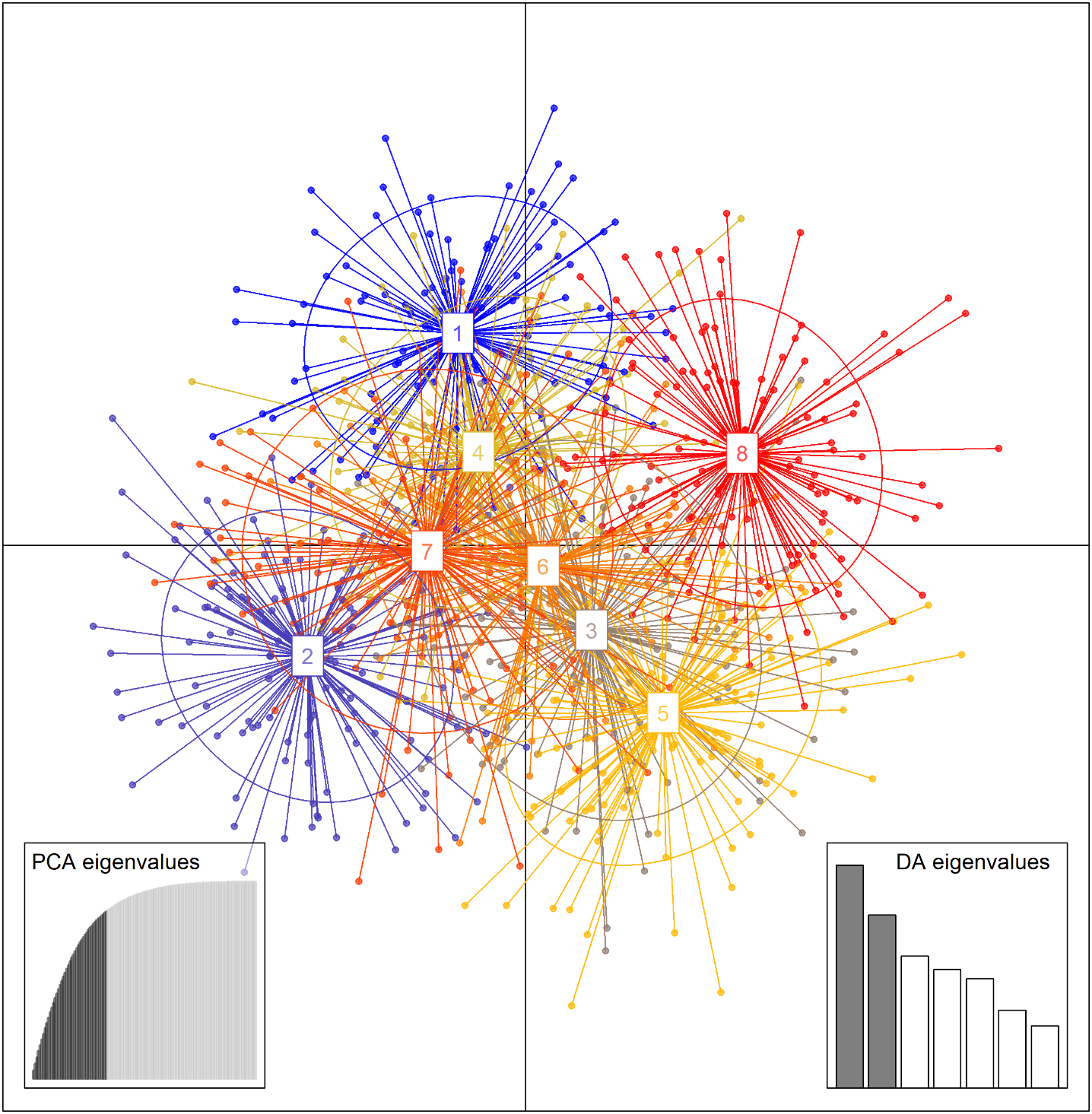

**Supplementary Figure 4.** Scatterplot of the first two Linear Discriminants (LD) showing genetic clusters (K=8) for 954 indigenous goats sampled in the three vegetation zones of Benin applying unsupervised Discriminant Analysis of Principal Components (DAPC). Each ellipse represents a priori cluster and each dot an individual.


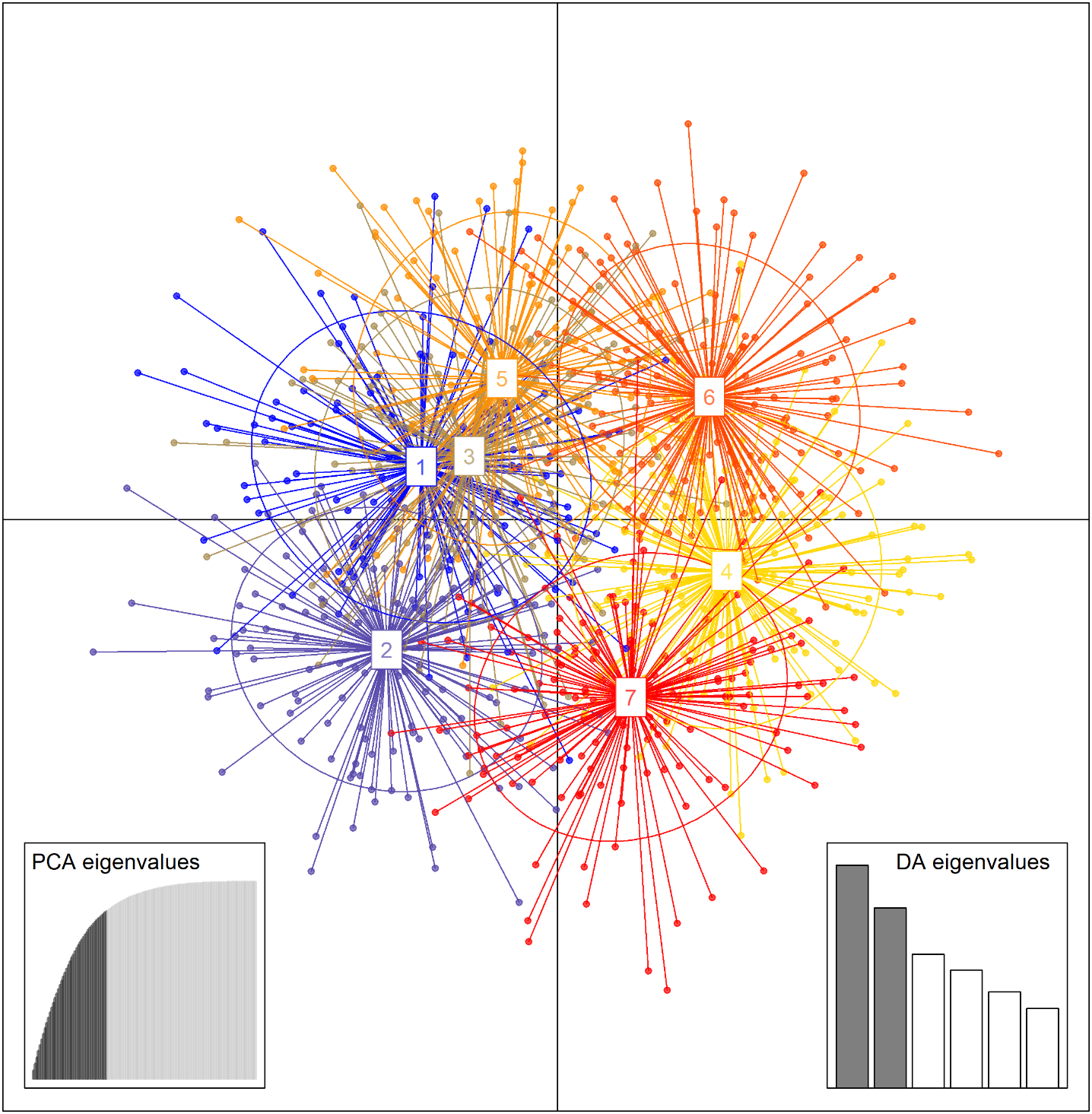


**Supplementary Figure 5.** Scatterplot of the first two Linear Discriminants (LD) showing genetic clusters (K=7) for 954 indigenous goats sampled in the three vegetation zones of Benin applying unsupervised Discriminant Analysis of Principal Components (DAPC). Each ellipse represents a priori cluster and each dot an individual.


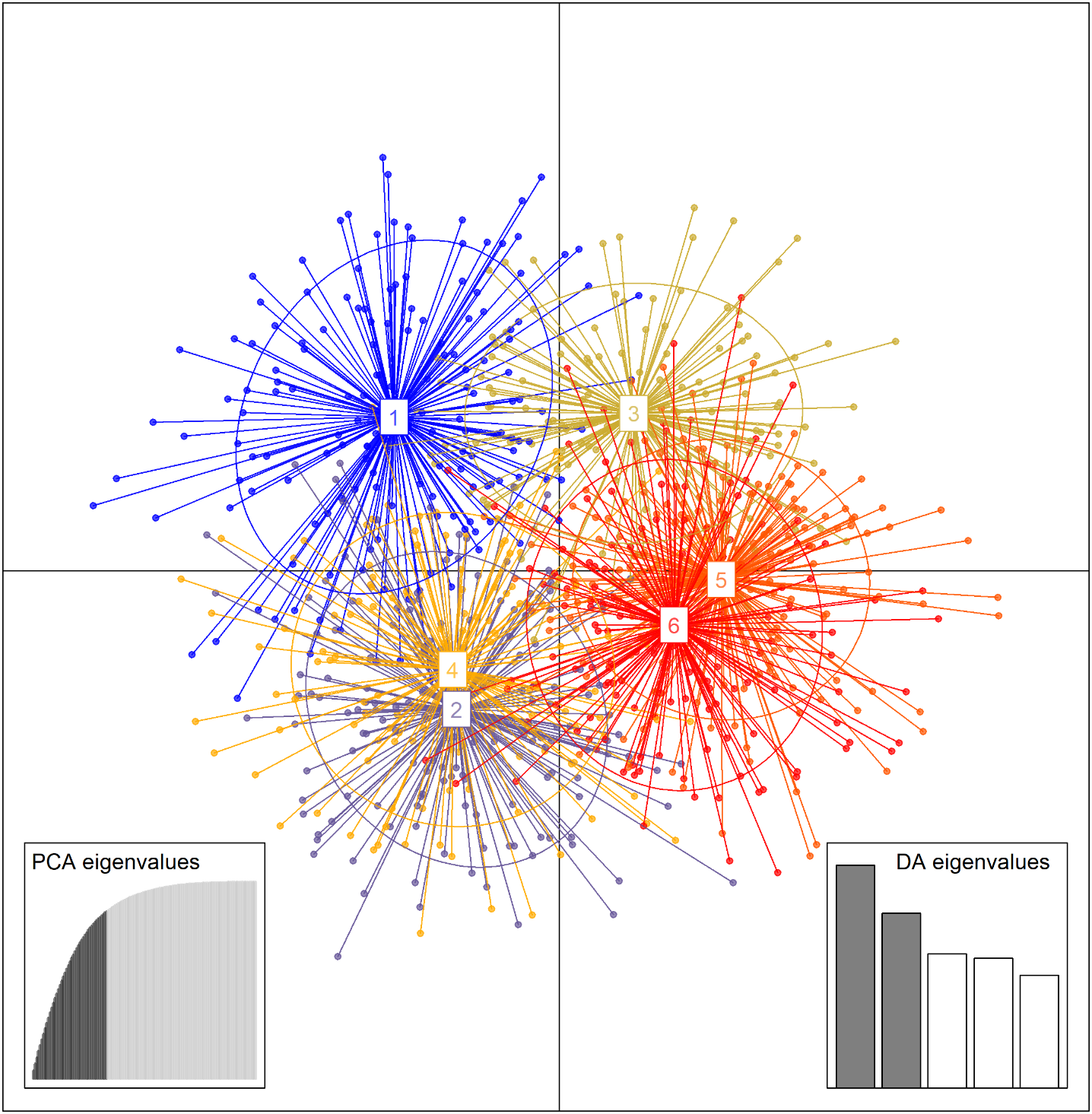


**Supplementary Figure 6.** Scatterplot of the first two Linear Discriminants (LD) showing genetic clusters (K=6) for 954 indigenous goats sampled in the three vegetation zones of Benin applying unsupervised Discriminant Analysis of Principal Components (DAPC). Each ellipse represents a priori cluster and each dot an individual.


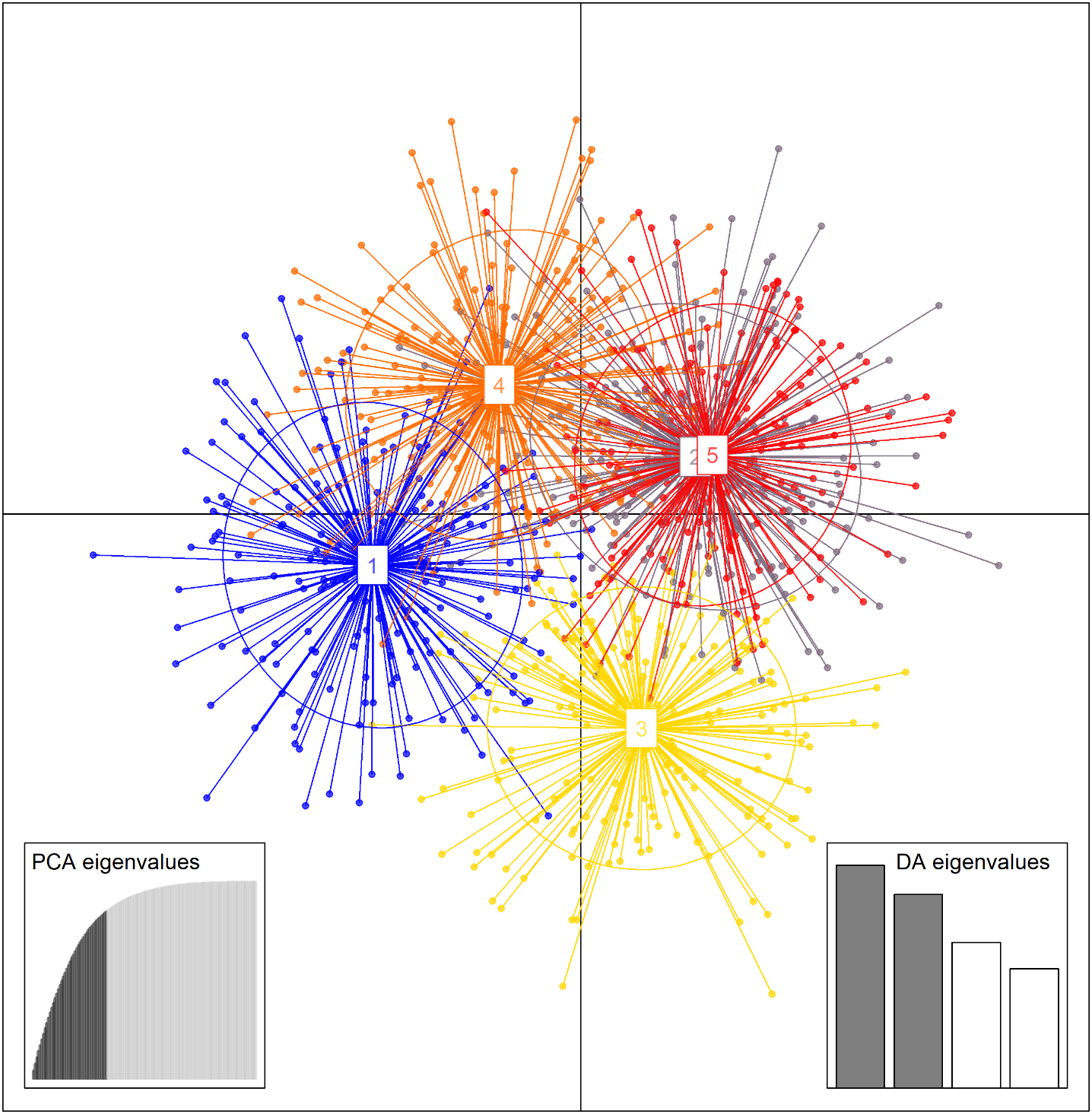


**Supplementary Figure 7.** Scatterplot of the first two Linear Discriminants (LD) showing genetic clusters (K=5) for 954 indigenous goats sampled in the three vegetation zones of Benin applying unsupervised Discriminant Analysis of Principal Components (DAPC). Each ellipse represents a priori cluster and each dot an individual.

# Supplementary Tables

**Supplementary Table 1.** Modal distribution of Delta K values

| **K** | **Reps** | **Mean LnP(K)** | **Stdev LnP(K)** | **Ln'(K)** | **\|Ln''(K)\|** | **Delta K** |
| --- | --- | --- | --- | --- | --- | --- |
| 1 | 3 | -34799.467 | 0.208167 | — | — | — |
| 2 | 3 | -34181.467 | 9.544283 | 618 | 507.43333 | 53.166209 |
| 3 | 3 | -34070.9 | 19.800758 | 110.56667 | 44.2 | 2.232238 |
| 4 | 3 | -34004.533 | 9.419306 | 66.366667 | 136.56667 | 14.498591 |
| 5 | 3 | -34074.733 | 44.57604 | -70.2 | 60.933333 | 1.366953 |
| 6 | 3 | -34084 | 27.528712 | -9.266667 | 462.86667 | 16.81396 |
| 7 | 3 | -34556.133 | 170.52675 | -472.13333 | 204.2 | 1.197466 |
| 8 | 3 | -35232.467 | 230.90821 | -676.33333 | 610.13333 | 2.64232 |
| 9 | 3 | -35298.667 | 918.80594 | -66.2 | 277.23333 | 0.301732 |
| 10 | 3 | -35087.633 | 275.42695 | 211.03333 | — | — |

Supplementary Table 2. Assignment proportion and repartition of the Beninese indigenous goat population estimated by Structure for K=2 (N=954)

|  | Assignment proportion (%) | Number of individuals in the population |
| --- | --- | --- |
| Ancestral goat population 1 | 50.20 | 479 |
| Ancestral goat population 2 | 49.80 | 475 |
| Total | 100 | 954 |
